# Intensive hog farming operations and self-reported health among nearby rural residents in Ottawa, Canada

**DOI:** 10.1186/1471-2458-9-330

**Published:** 2009-09-10

**Authors:** Paul J Villeneuve, Amira Ali, Laurel Challacombe, Sophie Hebert

**Affiliations:** 1 Dalla Lana School of Public Health, University of Toronto, Toronto, Ontario M5T 3M7, Canada; 2 Ottawa Public Health.100 Constellation Crescent Ottawa, Ontario K2G 6J8, Canada

## Abstract

**Background:**

In 2004, hog farming operations were introduced in the village of Sarsfield in the eastern part of Ottawa, Canada. This study evaluates the health-related quality of life (HRQOL), and the prevalence of respiratory conditions among adults and children who lived in proximity to this farm.

**Methods:**

A cross-sectional survey was administered to a random sample of residents from seven rural communities in the eastern part of Ottawa, Canada. We analyzed self-reported questionnaire data obtained from 723 adults and 285 children/adolescents. HRQOL was assessed using the SF-36 survey instrument, while data were also collected for sociodemographic characteristics, the prevalence of selected health conditions, and lifestyle related behaviours (e.g., smoking) of participants. Variations in self-reported health according to the residential distance to the hog farm were evaluated using logistic regression and analysis of variance methods.

**Results:**

For the most part, the prevalence of selected health conditions among adults and children was not associated with how far they lived from the farm. No associations were observed with migraines, respiratory conditions (asthma, rhinitis, sinusitis, and chronic bronchitis), and allergies. However, a higher prevalence of depression was noted among those who lived within 3 km of the farm relative to those who lived more than 9 km away (odds ratio = 2.01, 95% CI = 1.11, 3.65). Furthermore, individuals who lived closer to the IHF were more likely to worry about environmental issues such as water quality, outdoor and indoor smells, and air pollution. This level of worry also contributed to lower HRQOL scores for individuals who lived closer to the farm. It was also observed that the prevalence of depression was much higher among those who indicated a concern about environmental issues (18.2%) when compared to those who did not (8.0%).

**Conclusion:**

While our findings suggest that living in close proximity to an IHF may adversely affect HRQOL these should be interpreted cautiously due to a lack of direct measures of environmental exposures, and possible biases inherent in the use of self-reported health measures.

## Background

Over the last two decades there has been a marked change in hog farming operations in Canada that has mirrored that in other developed countries. Once predominated by small family owned farms, the productions of hogs is now characterized by large scale intensive farming operations that involve hundreds, if not thousands, of sows. These intensive farming operations can generate pollution and odours that may threaten both human health and the nearby environment. Pollution emitted into the air include hydrogen sulphide, ammonia, dusts, endotoxins as well as complex mixes of volatile organic compounds (VOCs) [1].

Numerous studies have demonstrated that the respiratory health of farm workers exposed to high levels of these pollutants can be adversely affected. Detailed reviews of these occupational studies have been published by Donham et al [2], and Olson et al [3]. Workers in hog confinement areas have been found to have a higher prevalence of cough, phlegm production, scratchy throat, runny nose, burning and watery eyes, headaches, chest tightness, shortness of breath, wheezing and muscle aches and pains [4]. Other respiratory conditions that may develop in these workers include asthma like syndrome and chronic bronchitis, and the exacerbation of asthma [1].

More recently, investigations have been undertaken to characterize health effects among nearby residents of hog and other livestock farms. Odours generated from these farms are of particular concern to these residents, and they have been shown to result in more tension, more depression, more anger, more fatigue and less vigour among those living closer to hog farming operations [5]. Other studies have also observed that the annoyance of those who live closer to these farms contributes to a lower quality of life [6-10]. Nearby residents of hog farms may also have concerns about the potential impact of farm exposures on their respiratory health. A study of 36 rural residents in Iowa found higher rates of respiratory symptoms, nausea, and burning eyes among those who lived within two mile radius of the far compared to those who lived further away [10]. More recently, a study of North Carolina residents found reduced quality of life, excesses of headaches, runny nose, sore throats, coughing diarrhoea, and burning eyes among those who lived in the vicinity of the hog operation [9]. In contrast, many studies have consistently shown that being raised on a farm is associated with reduced prevalence of hayfever, asthma and atopy when compared to being raised in rural non-farm environments or in urban areas. However, a higher prevalence of asthma outcomes has recently been observed among children living on farms where swine are raised (44.1%), despite lower rates of atopy and self-reported allergy [11]. This finding prompted the authors of this report to recommend for further population-based studies to investigate asthma outcomes in children who lived on such farms.

The evidence compiled to date is suggestive that emissions from intensive hog farming operations may adversely affect the health and mood of nearby residents. Many of these studies have had important limitations. The most common limitation has been relatively small sample sizes, which provide limited statistical power to compare prevalence estimates between areas for rare health conditions. Other limitations have included: the inability to adjust risk estimates for potential confounders, the lack of a standard instrument for the assessment of quality of life, a reliance on volunteers rather than a population based-sample, and the lack of direct measures of exposures.

A particular challenge to studying health effects among neighbouring residents of industrial hog farming operations is to derive objective measures of exposure and health outcomes. With self-reported data, associations may be found because an environmental exposure causes ill health. Alternatively, associations may be due in part, or in whole, to the increased reporting of symptoms or health conditions because the study participants are aware of the hazard. This phenomenon, termed "awareness bias" by Moffatt et al [12], necessitates that these two plausible explanations for observed associations be disentangled. While several studies of communities based hog farms have been undertaken, there has been little attempt to evaluate the role that awareness bias has played. An important exception to this is the work by Radon and colleagues who examined the respiratory health of residents who lived near animal feeding operations in Germany [13]. They demonstrated that self-reported respiratory symptoms were associated with increased annoyance of farm exposures, while clinical objective measures or respiratory function were not. This important finding underscores the importance of examining the possible role of awareness bias in studies reliant on self-reported data.

With this background in mind, we present our findings from a self-reported health survey administered to residents in the geographical vicinity of a large-scale intensive hog farm outside Ottawa, Canada. The aim of this study was to determine whether hog farming operations adversely affects the health of nearby residents. Our questionnaire (Additional file 1) was designed in such a manner that the effects of awareness bias could be characterized, and adjustment could be made for potential confounders. To our knowledge, this is the first Canadian community-based study that examines the possible health effects from hog farming in nearby residents.

## Methods

### Sarsfield intensive hog farm

The town of Sarsfield consists of approximately 275 homes, and 620 residents and is in the eastern part of Ottawa, Ontario. In 2004, and intensive hog farm was established just outside this town. The building application for the farm allowed for approximately 1,000 sows to be housed on the property. However, the size of the farm may increase as plans call for as many as 3,000 sows to be housed on the 667 acre site. Concerns about the possible environmental and human health impact of this farm expressed by residents resulted in decision of the City of Ottawa to fund this self-reported health survey.

### Overview of study design

The survey was designed to characterize the prevalence of several health conditions in the area of the farm shortly after farming operations had begun. With the planned expansion of the farm, it was hoped that this survey could provide valuable baseline data. In this cross-sectional study, we selected seven communities located in the eastern part of the city of Ottawa and in the general vicinity of intensive swine farm operations. To assess the HRQOL and the prevalence of health conditions in children and adults, a questionnaire was delivered to a sample of homes in these communities. The survey was presented as a general health survey, not as an assessment of the health effects of the IHF. This was done to minimize possible reporting biases by those concerned about the farm. The study was sponsored by Ottawa Public Health, and was approved by the City's ethics review board.

### Study population

We compiled a complete listing of eligible private households by using the city of Ottawa's property listings file. This file was regarded, for all intents and purposes, as being complete based on comparisons of dwelling counts for the study area using these listings to that of the 2001 Canadian census. The target population consisted of all residents of the communities that are located within twenty kilometres of the IHF, namely: Bearbrook, Cumberland, French Hill, Leonard, Navan, Sarsfield, and Vars. The total estimated population for these communities was approximately 8,100. Given the proximity of the IHF to the town of Sarsfield, all residents in this town were provided an opportunity to participate. Sampling ratios for the other communities were selected so as to compare prevalence of selected health conditions between communities while adhering to time and cost constraints. For communities other than Sarsfield, households were randomly chosen thus ensuring that each household within these areas had an equal probability of being sampled.

### Questionnaire

A questionnaire was developed to collect data on the general health of residents, and also incorporated questions on health conditions for which previous research had identified associations with hog farming operations. Pilot testing of the questionnaire was done on a sample of 25 individuals to ensure that it could be easily understood, and could be completed within 30 minutes. Questionnaires were made available in both English and French. Sociodemographic characteristics for adult participants were collected including age, sex, household income, educational attainment and occupation. The age and sex of each child resident in the home (if any) were collected from one participating adult member of the household.

Adult participants were also asked whether they had any of a number of health conditions that persisted for at least 6 months, and had been diagnosed by a health professional. This list consisted of several respiratory conditions including asthma, wheeze, chronic bronchitis, wheeze, sinusitis and rhinitis. Participants were also asked about whether they had been diagnosed with cardiovascular conditions, gastrointestinal disorders, or mental health conditions such as depression, anxiety, and migraines. Questionnaire data collected enabled us to classify individuals according to their cigarette smoking habits, and their body mass index. One adult household member provided information on behalf of the children about their general health status and the prevalence of respiratory conditions.

Health related quality of life (HRQOL) was assessed with the widely-used SF-36 survey instrument[14]. This survey instrument measures a person's ability to function while at work, at home, and in social situations. A standardized score with a range between 0 and 100 is calculated across 8 health domains, with a higher value reflecting better HRQOL.

To evaluate the extent of possible awareness bias, adult participants were asked to indicate from a pre-specified list of 18 items what had been sources of worry for them in the past year. IHF related worries included outdoor smells, air pollution, or water pollution. Finally, the survey included an open-ended question at the end whereby participants could provide any additional comments.

### Administration of survey

In October 2005, questionnaire packages were assembled and delivered to the selected households by a survey team of two individuals. The survey was presented as a general health questionnaire. The survey team visited each eligible home up to three times to establish contact. The visits were coordinated to ensure that the interview teams visited the homes at different times of the day, and if contact was not established during the initial visit, a weekend visit was made. On the third visit, if contact was not made, the questionnaire package was left in the mailbox of the home.

Both English and French language questionnaires were made available. Each adult member of the household was asked to complete a questionnaire, while one adult was asked to supply information for all children that resided in the home. Participants were asked to complete the questionnaire at their leisure, and return it with a postage paid envelope that was provided. We accepted all questionnaires returned by December 31, 2005.

In May 2006, a second SF-36 questionnaire was mailed to households that indicated they would be willing to participate in subsequent surveys. This allowed us to evaluate changes in health related quality of life that might exist due to seasonal variations. No analyses of the second survey are presented in this paper, largely because no appreciable differences in SF-36 scores were observed using a matched design approach.

Responses from the questionnaires were entered into a database using a double data entry method. Where discrepancies were found, manual resolution was used to ensure the proper value would be used in the analysis. The number of questionnaires for which manual resolution was undertaken was relatively small (approximately 30 questionnaires).

### Participation

Questionnaires were delivered to a total of 829 households, and of these, 37 refused to accept the materials. At least one questionnaire was completed and returned by mail by 419 (50.5%) households. Data were collected for 741 adults and 285 children. From these questionnaires, we excluded those (n = 7) where the residential address could not be ascertained and a further 11 for whom their age could not be ascertained. When compared to Canadian census data for the study region, survey participants were more likely to be female, and over the age of 45, and have French as their mother tongue. Rates of employment were similar between census and survey data for the area.

### Statistical methods

Descriptive statistics for each questionnaire item were generated to identify outliers or data anomalies. For each participant with available address, geographical information system (GIS) methods were used to calculate the distance between their homes and the IHF. We created a categorical variable to classify participants according to their residential distance to the hog farm. For adults, the distance categories were < 3, 3 - < 9, and ≥ 9 km. We choose these cutpoints for three important reasons. First, the selection of the cutpoints of <3, 3-<9 and 9> km grouped the number of participants into nearly equal groups (231, 236, and 256) thereby optimizing statistical power to compare disease prevalence rates across three distance groupings. Second, the study was initiated, in part, from concerns about the potential health concerns among residents of Sarsfield. The selection of a 3 km buffer essentially placed all residents of Sarsfield in the smallest distance category, while grouping participants from the other villages in the upper distance groupings. In particular, the mean distance between the residence and the IHF among Sarsfield participants was 2.1 km (Range 0.5 -3.1). As there were far fewer children than adults in the study, and given the prevalence of the health conditions under study, and the need to adjust for other covariates we opted to use two rather than three distance categories. The third reason for selecting a range of within 3 km was that recent research has demonstrated a high prevalence of reporting malodour among those who lived within 1.5 miles (2.4 km) from industrial swine operations [8]. In our study, we had data on a smaller number of children than adults, and therefore, lacked the statistical power to model these same three distance categories. Instead, we used the two groupings of <3, and ≥ 3 km.

Multivariable logistic regression was used to evaluate associations between residential distance to the hog farm, and the self-reported prevalence of various health conditions. Odds ratios generated from these regression models were adjusted for the possible confounding influence of other risk factors including: age, sex, cigarette smoking, and body mass index (BMI). BMI was treated as a potential confounding factor as several studies have found associations between it and respiratory symptoms, as well as mood disorders including depression. However, it made little difference to our measures of association and ultimately was dropped in the final models. We also examined the influence of socioeconomic status using household income on the risk estimates as previous work has shown that the validity of self-reported measures of health may vary by SES, and SES is associated with other determinants of health including diet, hygiene, and smoking. The standard errors of the odds ratios were adjusted for correlations arising from multiple questionnaires returned from the same household through the use of the method of generalized estimating equations. Statistical significance was assessed through inspection of the 95% confidence intervals of the odds ratios. We applied similar methods to compare the prevalence of childhood respiratory conditions.

Analysis of variance methods were used to compare SF-36 scores according to proximity to the IHF. The method of least squares was used to produce estimates for each of the SF-36 domains within each distance category adjusted for age, sex and income. To evaluate the possible role of awareness bias, stratified analysis was performed by grouping subjects according to whether they experienced any IHF related worries, or not.

## Results

The total number of households in the sampling frame within each community is presented in Table 1. Questionnaires were returned from 419 households, however, seven questionnaires were excluded because place of residence data were unavailable. Survey data were available for 734 adults, and 285 individuals less than 18 years of age. As mentioned before, age was not provided for 11 of the adult questionnaires and these questionnaires were dropped from subsequent analyses.

**Table 1:** Number of households and individuals who participated in general health survey, by community

**Community**	Number of Households*	**Households Approached**	**Participating Households**	**Household Participation Rate (%)**	**Adult Participants**	**Children (< 18 years of age)**
Bearbrook	189	31	11	35.5	23	4
Cumberland	1,357	158	84	53.2	149	50
French Hill	154	29	14	48.3	21	16
Leonard	53	32	17	53.1	32	11
Navan	747	170	89	52.4	156	89
Sarsfield	276	248	131	52.8	235	76
Vars	408	161	66	41.0	118	39

Overall	3,184	829	412	49.7	734	285

*based on property listing file maintained by the City of Ottawa

Sociodemographic characteristics according to distance from the hog farm for the adult participants are provided in Table 2. Approximately 15% of participants smoked cigarettes on a daily basis, while more than half of all adults (54.0%) were either overweight or obese. With respect to distance to the hog farm, 184 adults lived within 3 km from the IHF. Those who lived closer to the farm were more likely to be female, of lower educational attainment, and had a lower household income relative to those who lived further away.

**Table 2:** Sociodemographic characteristics of 723 adult participants of the Cumberland general health survey according to distance to farm

	**< 3 km**		**3 - < 9 km**		**≥ 9 km**	
	
**Characteristic**	**N**	**%**	**N**	**%**	**N**	**%**
Age-group						
18 - < 35	34	14.7	13	5.5	29	11.3
30 - < 50	85	36.8	104	44.1	112	43.8
45 - < 65	79	34.2	84	35.6	85	33.2
60 - < 75	29	10.4	21	8.9	21	8.2
≥ 75	9	3.9	14	5.9	9	3.5
Income (CDN$)						
≥ 80,000	69	29.9	117	49.6	114	44.5
50,000 - < 80,000	42	18.2	43	18.2	47	18.4
30,000 - < 50,000	34	14.7	15	6.4	25	9.8
15,000 - < 30,000	19	8.2	13	5.5	14	5.5
< 15,000	5	2.2	9	3.8	8	3.1
Prefer not to answer	62	26.8	39	16.5	48	18.8
Highest educational attainment						
No postsecondary degree	94	40.7	61	25.9	77	30.1
Trades certificate or diploma	45	19.5	22	9.3	28	10.9
Non-university certificate or diploma	47	20.4	54	22.9	75	29.3
University (below bachelor's degree)	8	3.5	13	5.5	10	3.9
Bachelor's degree	15	6.5	51	21.6	29	11.3
University graduate work	9	3.9	31	13.1	22	8.6
Unknown	13	5.6	4	1.7	15	5.9
Smoking						
Daily	36	15.6	30	12.7	44	17.2
Occasional	5	2.2	7	3.0	17	6.6
Non-smoker	187	80.9	199	84.3	192	75.0
Unknown	3	1.3	0	0.0	3	1.2
Regular smoker inside house						
Yes	193	83.5	213	90.3	221	86.3
No	37	16.0	22	9.3	32	12.5
Unknown	1	0.4	1	0.4	3	1.2
Body mass index						
Underweight (< 18.5)	3	1.3	7	3.0	5	2.0
Healthy weight (18.5 - < 25)	80	34.6	94	39.8	104	40.6
Overweight (25 - < 30)	77	33.3	78	33.1	96	37.5
Obese (30+)	48	20.8	49	20.8	42	16.4
Unknown	23	10.0	8	3.4	9	3.5
Men	100	43.3	130	55.1	138	53.9
Women	131	56.7	106	44.9	118	46.1
Total	231	100.0	236	100.0	256	100.0

For the most part, there were no statistically significant associations found between proximity to the IHF and the prevalence of several respiratory conditions (Table 3). An increased prevalence of chronic bronchitis was reported by individuals who lived within 3 kilometres relative to those who lived ≥ 9 kilometres however, the precision of the estimate is poor given the relatively small number of cases away (OR = 2.12, 95% CI = 0.82, 5.47).

**Table 3:** Self-reported prevalence of selected respiratory system conditions among 723 adult participants by the distance between their residence and the intensive hog farm.

**Health condition**	**Distance to IHF**	**Prevalence**		**Odds ratio^A ^and 95% CI**			**Odds ratio^B ^and 95% CI**		
		
		**N**	**%**	**OR**	**LL**	**UL**	**OR**	**LL**	**UL**
Asthma	≥ 9 km	29	11.3	1.0			1.0		
	3 - < 9 km	20	8.5	0.74	0.38	1.42	0.77	0.40	1.50
	<3 km	22	9.5	0.82	0.43	1.56	0.80	0.41	1.52
									
Asthma symptoms in last year	≥ 9 km	23	9.0	1.0			1.0		
	3 - < 9 km	12	5.1	0.56	0.26	1.19	0.55	0.26	1.19
	<3 km	20	8.6	0.96	0.48	1.90	1.05	0.52	2.10
									
Asthma medication	≥ 9 km	25	18.6	1.0			1.0		
In last year	3 - < 9 km	18	7.6	0.78	0.40	1.51	0.78	0.39	1.55
	<3 km	23	10.0	1.11	0.53	1.95	1.11	0.57	2.15
									
Wheeze	≥ 9 km	43	16.8	1.0			1.0		
	3 - < 9 km	34	14.4	0.84	0.49	1.43	0.91	0.52	1.56
	<3 km	43	18.6	1.14	0.67	1.94	1.10	0.63	1.91
									
Rhinitis	≥ 9 km	40	15.6	1.0			1.0		
	3 - < 9 km	22	9.3	0.56	0.31	1.01	0.55	0.31	0.99
	<3 km	35	15.2	0.96	0.57	1.63	0.96	0.57	1.63
									
Nasal allergies	≥ 9 km	63	24.6	1.0			1.0		
	3 - < 9 km	51	21.6	0.90	0.56	1.42	0.88	0.55	1.39
	<3 km	52	22.5	0.88	0.56	1.38	0.89	0.57	1.40
									
Chronic Bronchitis	≥ 9 km	8	3.1	1.0			N.E.		
	3 - < 9 km	10	4.2	1.34	0.52	3.46			
	<3 km	15	6.5	2.12	0.82	5.47			
									
Sinusitis	≥ 9 km	29	11.3	1.0			1.0		
	3 - < 9 km	19	8.1	0.68	0.37	1.26	0.67	0.36	1.24
	<3 km	33	14.3	1.29	0.75	2.20	1.34	0.78	2.30

N.E. = Not estimable

^A ^Odds ratios were adjusted for age, sex and smoking status; generalized estimating equations were used to adjust standard errors for multiple questionnaires completed from the same household

^B ^Odds ratios were adjusted for age, sex, smoking status and household income; generalized estimating equations were used to adjust standard errors for multiple questionnaires completed from the same household

Adults who lived within 3 km of the IHF were nearly twice as likely to report having been diagnosed with depression compared to those who lived at least 9 kilometres from the farm (OR = 2.01, 95% CI = 1.11, 3.65) (Table 4). However, it is important to note the absence of a dose response relationship as the risk estimate for those who lived 3-<9 km, had approximately the same prevalence as those who lived within 3 km from the farm. There were no statistically significant associations found with proximity to the IHF for several other health conditions examined such as migraines, and gastrointestinal disorders.

**Table 4:** Self-reported prevalence of selected health conditions among 723 adult participants, by distance between residence and the intensive hog farm.

**Health condition**	**Distance to IHF**	**Prevalence**		**Odds ratio^A ^and 95% CI**			**Odds ratio^B ^and 95% CI**		
		
		**N**	**%**	**OR**	**LL**	**UL**	**OR**	**LL**	**UL**
High blood pressure	≥ 9 km	36	14.1	1.0			1.0		
	3 - < 9 km	46	19.5	1.39	0.83	2.31	1.45	0.87	2.43
	<3 km	40	17.3	1.29	0.76	2.19	1.27	0.74	2.20
									
Depression	≥ 9 km	21	8.2	1.0			1.0		
	3 - < 9 km	19	8.1	0.97	0.48	1.92	0.93	0.47	1.86
	<3 km	34	14.7	1.91	1.05	3.46	2.01	1.11	3.65
									
Migraines	≥ 9 km	30	11.7	1.0			1.0		
	3 - < 9 km	27	11.4	0.99	0.56	1.74	0.96	0.55	1.65
	<3 km	36	15.6	1.37	0.81	2.30	1.48	0.87	2.49
									
Anxiety	≥ 9 km	30	11.7	1.0			1.0		
	3 - < 9 km	26	11.0	0.88	0.51	1.54	0.87	0.50	1.53
	<3 km	32	13.8	1.18	0.70	2.00	1.18	0.68	2.03
									
Arthritis	≥ 9 km	57	22.3	1.0			1.0		
	3 - < 9 km	49	20.8	0.79	0.49	1.28	0.82	0.50	1.33
	<3 km	59	25.5	1.18	0.75	1.86	1.13	0.70	1.83
									
Eczema	≥ 9 km	26	10.2	1.0			1.0		
	3 - < 9 km	25	10.6	1.11	0.62	1.97	1.02	0.57	1.83
	<3 km	15	6.5	0.59	0.30	1.16	0.63	0.31	1.25

N.E. = Not estimable

^A ^Odds ratios were adjusted for age, sex and smoking status; generalized estimating equations were used to adjust standard errors for multiple questionnaires completed from the same household

^B ^Odds ratios were adjusted for age, sex, smoking status and household income; generalized estimating equations were used to adjust standard errors for multiple questionnaires completed from the same household

Comparison of the prevalence of selected health conditions among children, as reported by their parents is provided in Table 5. Due to the much smaller number of responses obtained for children only two distance categories were used (<3 and ≥ 3 km). No statistically significant increases in prevalence were observed for asthma, wheeze, hayfever, allergies or runny nose (p > 0.05).

**Table 5:** Prevalence of respiratory conditions among 275 children/adolescents* as reported by their parents, by distance between residence and the intensive hog farm

**Health condition**	**Distance to IHF**	**Prevalence**		**Odds ratio^A ^and 95% CI**			**Odds ratio^B ^and 95% CI**		
		
		**N**	**%**	**OR**	**LL**	**UL**	**OR**	**LL**	**UL**
Asthma (lifetime)	≥ 3 km	44	21.7	1.0			1.0		
	< 3 km	17	23.6	1.09	0.51	2.35	1.06	0.48	2.37
									
Asthma (current)	≥ 3 km	23	11.3	1.0			1.0		
	< 3 km	10	13.9	1.27	0.54	2.98	1.25	0.54	2.94
									
Wheeze	≥ 3 km	44	21.7	1.0			1.0		
	< 3 km	21	29.2	1.53	0.76	3.06	1.44	0.72	2.91
									
Hayfever	≥ 3 km	20	9.9	1.0			1.0		
	< 3 km	7	9.7	0.96	0.37	2.54	0.98	0.34	2.84
									
Allergies	≥ 3 km	56	27.6	1.0			1.0		
	< 3 km	25	34.7	1.38	0.73	2.61	1.44	0.74	2.78
									
Runny nose in absence of flu	≥ 3 km	60	29.6	1.0			1.0		
	< 3 km	25	34.7	1.26	0.64	2.45	1.24	0.63	2.43

^A ^Odds ratios were adjusted for age, sex and smoking status; generalized estimating equations were used to adjust standard errors for multiple questionnaires completed from the same household

^B ^Odds ratios were adjusted for age, sex, smoking status and household income; generalized estimating equations were used to adjust standard errors for multiple questionnaires completed from the same household

**Table 6:** Least squares age, sex and income adjusted SF-36 scores* among 723 adult participants according to distance to the intensive hog farm (IHF), and whether they had IHF-related worries

**DomainM**	**No IHF worries (n = 555)** **Distance in (km)**				**≥ 1 IHF worry (n = 168)** **Distance in km**				**All participants (n = 723)** **Distance in km**			
	<3	3 - < 9	≥ 9	p	<3	3-<9	≥ 9	p	<3	3 - <9	≥ 9	p
PF	77.9	80.0	82.0	0.22	72.5	79.8	76.9	0.06	76.3	80.1	81.1	0.02
RP	73.4	76.7	78.1	0.52	62.5	68.2	67.2	0.17	70.0	75.1	76.1	0.06
BP	65.8	70.5	68.6	0.18	54.3	61.3	59.5	0.12	62.7	69.3	67.1	<0.01
GH	72.5	71.5	71.5	0.73	59.3	64.1	58.7	0.37	68.8	70.5	69.3	0.68
VI	61.6	62.0	61.8	0.98	49.0	50.9	51.0	0.87	58.6	60.5	60.0	0.46
SF	79.0	83.9	85.3	0.02	62.5	66.8	68.7	0.60	74.9	81.4	82.6	<0.01
RE	80.9	82.1	83.5	0.76	68.3	73.9	76.6	0.26	76.7	81.2	82.2	0.07
MH	76.7	78.4	78.4	0.69	69.0	77.0	74.6	0.06	74.1	78.1	77.6	0.02
PCS	46.5	47.6	47.7	0.62	42.6	45.1	44.3	0.12	45.5	47.2	47.1	0.06
MCS	51.2	52.0	52.2	0.60	45.6	48.0	48.3	0.29	49.6	51.4	51.5	0.04

PF = Physical Functioning; RP = Role Play; BP = Body Pain; GH = General Health; VI = Vitality

SF = Social functionsing; RE = Role emotional; MH = Mental Health; PCS = Physical component score; MCS = Mental component score

* Based on ANOVA methods; excludes those with missing age or distance measures

In table 6, we present HRQOL scores for each of the domains of the SF-36 in relation to distance from hog farm as well as whether individuals' indicated any worries related to the farm. On average, HRQOL scores were higher for those who lived further than 3 km from the IHF (Table 6). Differences in the adjusted mean scores between those who lived within 3 km of the farm and those who lived either 3-<9 km, or ≥ 9 km were often larger than 5 units. Previous authors have suggested that such a magnitude of change may be interpreted as a clinically important difference. The difference in HRQOL scores between those who lived closer to the farm and those who lived further away was generally larger among those who reported having an IHF-related worry. Among those with no IHF related worries, HRQOL were usually lower among those who lived closer to the farm. However, a statistically significant association was noted for only one of the domains (Social Functioning) in these individuals.

## Discussion

Our study found no statistically significant associations between self-reported prevalence of respiratory health conditions and residential proximity to the intensive hog farm for either children or adults. On the other hand, individuals who lived closer to the farm reported a reduced HRQOL, and an increased prevalence of depression. Our findings for these two outcomes are consistent with previous studies that evaluated the health of residents who neighbour large scale farming operations. In particular, a previous study that examined respiratory health among residents who lived near hog farming operation in North Carolina found that those who lived within a 2 mile radius of hog farms were more likely to report headaches and have a poorer quality of life when compared to those who lived near cattle farms, at least 2 miles from other farming operations[9] This US based study also found that those who lived with 2 miles of a hog farm were more likely to report symptoms of runny nose, a sore throat, excessive coughing and symptoms associated with skin an eye irritation. The climates of Ottawa and North Carolina vary considerably, with residents in Ottawa more likely to spend a greater portion of their time indoors during the winter months. The interpretation of our findings should also carefully take into account some of the limitations of the survey, and additionally, the role of awareness bias. A more detailed treatise of these issues is provided below.

Some important strengths of our study included the ability to construct a sampling list of virtually all households in the targeted communities, a relatively large number of participants (723 adults and 285 children), and the use of standardized instrument (SF-36) to evaluate health related quality of life. We used wording similar to recently conducted national health surveys. By so doing, we employed questions that had previously been validated in both English and French and allowed for a comparison of disease prevalence estimates to those for the province of Ontario.

A limitation of our study was the modest participation rates of those who were asked to complete the questionnaires. Roughly one half of households invited to participate did so. We expended considerable effort to maximize participation. This included: contacting community groups to inform them of the study, inviting members of community groups to assist with the delivery of the questionnaires, a cover letter prepared by the regional medical officer of health advertising the study, door-to-door delivery of questionnaire packages, publicizing the study in local newspapers, community centres and on the internet, providing contact information for individuals who wanted additional information, providing a postage paid envelope for questionnaires to be returned, and mailing postcards to contacted homes to remind individuals to return the survey. Participation may have been compromised in part due to the property assessment notices that were delivered to residents shortly before the survey was delivered. Dramatic increases in property values, common in this assessment, may have discouraged participation in a health survey sponsored by a local level of government. Comparisons of the survey population to data for the study region based on census data indicate that women, and those over 45 are overrepresented in the study sample. This likely reflects in part, the fact that such individuals were more likely to be at home when the initial visit was made by the survey team. The lower response rates make it difficult to generalize the self-reported health measures obtained from the survey to the entirety of the target population. Given that participation varied between communities, and hence distance to the IHF, it is possible that our presented associations may be biased.

Comparisons of sociodemographic characteristics between those who lived within 3 km of the IHF to those who lived further away revealed important differences. Namely, those in closer proximity to the hog farm, had lower household incomes, and lower educational attainment. Being in a low socioeconomic group is a well recognized risk factor for depression[15]. This may be due to factors such as perceived low social status, cultural factors, financial problems, stressful environments, social isolation, and greater daily stress.

Unfortunately, this study did not have any direct exposure measures. It was assumed that person who lived < 3 km from the farm were more highly exposed than others. Variations in exposure may have existed due to differences in elevation, meteorology (wind), time spent indoors or outdoors, availability of air conditioning and the amount of time spent at home. Further efforts to enhance the characterization of these exposures at a more refined spatial level would enhance the ability to detect associations. Despite this limitation, it is important to note that this study was conducted as a first step to explore whether excesses in several health conditions were evident in the town of Sarsfield which was closest to the farm. Further, it was a valuable undertaking to gauge the extent to which residents in this community were willing to participate in an ongoing health assessment. Stronger studies that would include objective outcome measures determine through clinical testing or biological sampling would necessitate an increased level of commitment to participate among residents. Our modest levels of participation suggest that undertaking such a study would prove difficult.

## Conclusion

Our findings suggest that living in close proximity to an IHF may adversely affect residents' health-related quality of life. These findings should be interpreted cautiously due to a lack of direct measures of environmental exposures, participation bias, and limitations of using self-reported measures of health status. Further research in this population should incorporate environmental monitoring data.

## Competing interests

The authors declare that they have no competing interests.

## Authors' contributions

PV was the principal investigator of the study and participated in the design of the study, the development of the questionnaire, the statistical analysis of the data, and the writing of the paper. AA participated in the design of the study, assisted in the development of the questionnaire and contributed to the writing and interpretation of the findings. LC performed statistical programming, assisted in the writing of the manuscript and the interpretation of the results. SH was the coordinator of the study and assisted in the training of interviewers, data entry, some statistical analyses, and assistance in the preparation of the manuscript. All authors read and approved the final manuscript.

## Pre-publication history

The pre-publication history for this paper can be accessed here:



## Supplementary Material

Additional file 1 **Cumberland_english_q**. A copy of the questionnaire used in the study Click here for file

## References

[B1] Von Essen S, Donham K (1999). Illness and Injury in animal confinement workers. Occupational Medicine.

[B2] Donham KJ (2000). The concentration of swine production. Effects on swine health, productivity, human health, and the environment. The Veterinary clinics of North America.

[B3] Olson DK, Bark SM (1996). Health hazards affecting the animal confinement farm worker. Aaohn J.

[B4] Donham KJ (1990). Health effects from work in swine confinement buildings. American journal of industrial medicine.

[B5] Schiffman SS, Miller EA, Suggs MS, Graham BG (1995). The effect of environmental odors emanating from commercial swine operations on the mood of nearby residents. Brain Res Bull.

[B6] Nimmermark S (2004). Odour influence on well-being and health with specific focus on animal production emissions. Ann Agric Environ Med.

[B7] Radon K, Peters A, Praml G, Ehrenstein V, Schulze A, Hehl O, Nowak D (2004). Livestock odours and quality of life of neighbouring residents. Ann Agric Environ Med.

[B8] Wing S, Horton RA, Marshall SW, Thu K, Tajik M, Schinasi L, Schiffman SS (2008). Air pollution and odor in communities near industrial swine operations. Environmental health perspectives.

[B9] Wing S, Wolf S (2000). Intensive livestock operations, health, and quality of life among eastern North Carolina residents. Environmental health perspectives.

[B10] Thu K, Donham K, Ziegenhorn R, Reynolds S, Thorne PS, Subramanian P, Whitten P, Stookesberry J, Reynolds S (1997). A control study of the physical and mental health of residents living near a large-scale swine operation. J Agric Safety Health.

[B11] Merchant JA, Naleway AL, Svendsen ER, Kelly KM, Burmeister LF, Stromquist AM, Taylor CD, Thorne PS, Reynolds SJ, Sanderson WT (2005). Asthma and farm exposures in a cohort of rural Iowa children. Environmental health perspectives.

[B12] Moffatt S, Mulloli TP, Bhopal R, Foy C, Phillimore P (2000). An exploration of awareness bias in two environmental epidemiology studies. Epidemiology.

[B13] Radon K, Schulze A, Ehrenstein V, van Strien RT, Praml G, Nowak D (2007). Environmental exposure to confined animal feeding operations and respiratory health of neighboring residents. Epidemiology.

[B14] Ware JE, Sherbourne CD (1992). The MOS 36-item short-form health survey (SF-36). I. Conceptual framework and item selection. Medical care.

[B15] Everson SA, Maty SC, Lynch JW, Kaplan GA (2002). Epidemiologic evidence for the relation between socioeconomic status and depression, obesity, and diabetes. Journal of psychosomatic research.

