# Supplementary material for: Intensive hog farming operations and self-reported health among nearby rural residents in Ottawa, Canada
Source: BMC Public Health. 2009 Sep 10;9:330. doi: 10.1186/1471-2458-9-330 (PMC2749827; doi:10.1186/1471-2458-9-330)
Supplement: Additional file 1 — Cumberland_english_q. A copy of the questionnaire used in the study [file 1471-2458-9-330-S1.pdf]

Ottawa

Sponsored by Ottawa Public Health

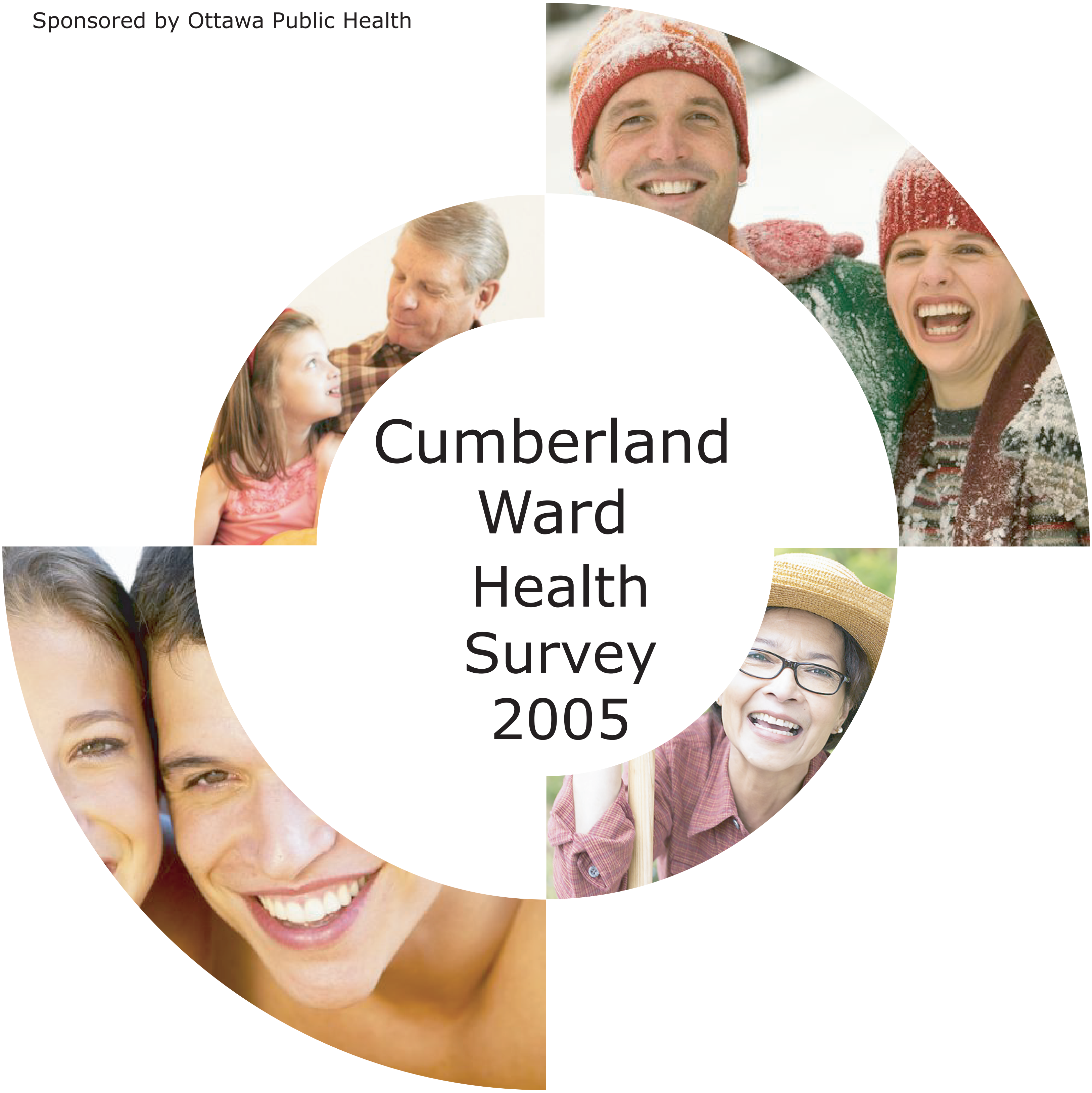

Cumberland Ward Health Survey 2005

Survey conducted by

EPISTREAM CONSULTING INC.

ABOUT THIS QUESTIONNAIRE

This questionnaire is part of the City of Ottawa's efforts to monitor the health of residents in Cumberland Ward. We greatly value your participation in this study and we hope that you find this questionnaire interesting to complete. The questionnaire is intended for household members who are 18 years of age or older. We ask that each resident above this age complete the questionnaire. Your answers are extremely important to us as they will be used to describe the health of your community and to monitor changes in the health of your community over time.

Please remember that your participation is voluntary, and that you may skip over any questions that you prefer not to answer.

Most questions can be answered by placing an 'X' in the appropriate box, or by circling the number or letter beside the most appropriate answer. For example,

1. What is your sex? ☐ Male ☒ Female

2. In general, would you say your health is:

Excellent	Very Good	Good	Fair	Poor
☐	☒	☐	☐	☐

Some questions require you to respond by providing a number. Please print your answer or best guess clearly within the space provided. For example,

3. How old are you? 26 years of age

Some questions require more than one response. Be sure to mark all items that apply. For example,

4. Do you have asthma?

☒ YES ☐ NO

If **YES**, how old were you when you were first diagnosed with asthma?

14 years

If you have any questions about this questionnaire, please feel free to call us at 613-580-2424, extension 14117.

Please return this questionnaire in the provided postage-paid envelope by November 15, 2005.

Thank You!

CUMBERLAND

CONTACT INFORMATION

We ask that you provide your name, phone number, and address in the space below. This information will be separated from the responses you have provided to the questionnaire and securely stored on site at the offices of Ottawa Public Health. This information will only be used by the study coordinator (if needed) to clarify some of your responses.

May we contact you for these specific reasons? ❑ YES ❑ NO

Name: ___________________________________

Address: ___________________________________

____

____

Postal Code: ___________________________________

Telephone: ( ) _____________________________ Type: ❑ Home ❑ Cell ❑ Work

( ) _____________________________ Type: ❑ Home ❑ Cell ❑ Work

Email: ___________________________________

Date: 2005 / ______ / ______

Year Month Day

If you need assistance to complete the questionnaire, or if you have any questions, please do not hesitate to call the study coordinator at Ottawa Public Health at 613-580-2424, extension 14117.

Please note: If you would like to keep a copy of the questionnaire for your own records, please make a duplicate BEFORE you mail it in. Once we have received your questionnaire, we will not be able to release it back to you.

Section A

In this first part of the questionnaire, we ask you to describe characteristics about your household and some general information about you.

1. How long have you lived at your current address? ____ years OR ____ months

2. How many people usually live in your household (this includes children who live at home on a part-time basis)? ____ people

3. How many household members are:
   a. Less than 18 years of age? ____ member(s)
   b. 18 years of age or older? ____ member(s)

4. How old are you? ____ years of age

5. What is your sex? ❑ Male ❑ Female

6. What is your height? ____ feet and ____ inches OR ____ centimetres

7. What is your weight? ____ lbs OR ____ kg

8. How many days in the last twelve months were you away from home on overnight trips or vacations?
   a. Less than 14 days
   b. 14 to 27 days
   c. 28 to 55 days
   d. 56 to 111 days
   e. 112 days or more

9. How many hours, on average, do you spend at home on each given day?
   a. Monday _____ hours
   b. Tuesday _____ hours
   c. Wednesday_____ hours
   d. Thursday_____ hours
   e. Friday _____ hours
   f. Saturday_____ hours
   g. Sunday _____ hours

10. How often do heavy vehicles (for example, trucks or buses) pass within 300 meters (or 3 blocks or 1000 feet) of your house?
    a. Less than 10 times an hour
    b. 10 to 49 times an hour
    c. 50 to 99 times an hour
    d. 100 times an hour or more
    e. Don't know

11. How often do cars pass within 100 meters (or 300 feet or 1 block) of your house?
    a. Less than 10 times an hour
    b. 10 to 49 times an hour
    c. 50 to 99 times an hour
    d. 100 times an hour or more
    e. Don't know

Section B

These first questions are about your health now and your current daily activities. Please answer every question by placing an 'X' in the appropriate box.

If you are unsure about how to answer a question, please give the best answer you can.

1. How would you rate your health, in general, now?

Excellent	Very Good	Good	Fair	Poor
▼ ❑	▼ ❑	▼ ❑	▼ ❑	▼ ❑

2. Compared to one year ago, how would you rate your health in general now?

Much better now than one year ago	Somewhat better now than one year ago	About the same as one year ago	Somewhat worse now than one year ago	Much worse now than one year ago
▼ ❑	▼ ❑	▼ ❑	▼ ❑	▼ ❑

3. The following questions are about activities you might do during a typical day. Does your health now limit you in these activities? If so, how much?

	Yes, limited a lot	Yes, limited a little	No, not limited at all
a. Vigorous activities, such as running, lifting heavy objects, participating in strenuous sports	❑	❑	❑
b. Moderate activities, such as moving a table, pushing a vacuum cleaner, bowling, or playing golf	❑	❑	❑
c. Lifting or carrying groceries	❑	❑	❑
d. Climbing several flights of stairs	❑	❑	❑
e. Climbing one flight of stairs	❑	❑	❑
f. Bending, kneeling, or stooping	❑	❑	❑
g. Walking more than one mile (1.6 kilometers)	❑	❑	❑
h. Walking several blocks (more than 100 meters)	❑	❑	❑
i. Walking one block	❑	❑	❑
j. Bathing or dressing yourself	❑	❑	❑

4. During the past 4 weeks, have you had any of the following problems with your work or other regular daily activities as a result of your physical health?

	YES	NO
a. Cut down on the amount of time you spent on work or other activities	❑	❑
b. Accomplished less than you would like	❑	❑
c. Were limited in the kind of work or other activities	❑	❑
d. Had difficulty performing the work or other activities (for example, it took extra effort)	❑	❑

5. During the past 4 weeks, have you had any of the following problems with your work or other regular daily activities as a result of any emotional problems (such as feeling depressed or anxious)?

	YES	NO
a. Cut down on the amount of time you spent on work or other activities	❑	❑
b. Accomplished less than you would like	❑	❑
c. Didn't do work or other activities as carefully as usual	❑	❑

6. During the past 4 weeks, to what extent has your physical health or emotional problems interfered with your normal social activities with family, friends, neighbors, or groups?

Not at all	Slightly	Moderately	Quite a bit	Extremely
❑	❑	❑	❑	❑

7. How much bodily pain have you had during the past 4 weeks?

None	Very mild	Mild	Moderate	Severe	Very severe
❑	❑	❑	❑	❑	❑

8. During the past 4 weeks, how much did pain interfere with your normal work (including both work outside the home and housework)?

Not at all	Slightly	Moderately	Quite a bit	Extremely
❑	❑	❑	❑	❑

9. These questions are about how you feel and how things have been with you during the past 4 weeks. For each question, please give the one answer that comes closest to the way you have been feeling. How much of the time during the past 4 weeks...

	All of the time	Most of the time	Some of the time	A little of the time	None of the time
a. Did you feel full of pep?	❑	❑	❑	❑	❑
b. Have you been very nervous?	❑	❑	❑	❑	❑
c. Have you felt so down in the dumps that nothing could cheer you up?	❑	❑	❑	❑	❑
d. Have you felt calm and peaceful?	❑	❑	❑	❑	❑
e. Did you have a lot of energy?	❑	❑	❑	❑	❑
f. Have you felt downhearted and depressed?	❑	❑	❑	❑	❑
g. Did you feel worn out?	❑	❑	❑	❑	❑
h. Have you been happy?	❑	❑	❑	❑	❑
i. Did you feel tired?	❑	❑	❑	❑	❑

10. During the past 4 weeks, how much of the time has your physical health or emotional problems interfered with your social activities (like visiting with friends, relatives, etc.)?

All of the time	Most of the time	Some of the time	A little of the time	None of the time
❑	❑	❑	❑	❑

11. How TRUE or FALSE is each of the following statements for you?

	Definitely true	Mostly true	Don't know	Mostly false	Definitely false
a. I seem to get sick a little easier than other people	❑	❑	❑	❑	❑
b. I am as healthy as anybody I know	❑	❑	❑	❑	❑
c. I expect my health to get worse	❑	❑	❑	❑	❑
d. My health is excellent	❑	❑	❑	❑	❑

Section C

In this section we ask about certain chronic conditions and/or recent symptoms that you may have had. When answering the questions, please remember that we are interested in "long-term" conditions that have lasted (or are expected to last) at least 6 months, and that have been diagnosed by a health professional.

1. Have you had wheezing or whistling in your chest at any time in the last 12 months?

   ❑ YES ❑ NO

2. Do you have asthma?

   ❑ YES ❑ NO If **NO**, go to Question 6.

   If **YES**, how old were you when you were first diagnosed with asthma?

   _____ years

3. Have you had any asthma symptoms or asthma attacks in the last 12 months?

   ❑ YES ❑ NO

   If **YES**, during which months of the year did you have the attacks (please check all that apply)?

❑ January	❑ February	❑ March	❑ April
❑ May	❑ June	❑ July	❑ August
❑ September	❑ October	❑ November	❑ December

4. In the past twelve months, have you visited an emergency room department because of your asthma?

   ❑ YES ❑ NO

5. In the past twelve months, have you taken any medicine for asthma such as inhalers, nebulizers, pills, liquids, or injections?

   ❑ YES ❑ NO

6. **REMEMBER, we are interested in conditions diagnosed by a health professional.**

Do you have any of the following conditions? If **Yes**, please provide the age when you were first told about this condition.

	Yes	No	Don't Know	If **Yes**, Age When Told
a. Food allergies	❑	❑	❑	_____ years
b. Nasal allergies including hay fever	❑	❑	❑	_____ years
c. Multiple chemical sensitivities	❑	❑	❑	_____ years
d. Migraine headaches	❑	❑	❑	_____ years
e. Urinary incontinence	❑	❑	❑	_____ years
f. Diabetes (other than during pregnancy)	❑	❑	❑	_____ years
g. Depression	❑	❑	❑	_____ years
h. Anxiety	❑	❑	❑	_____ years
i. Arthritis	❑	❑	❑	_____ years
j. Sinusitis (sinuses that are infected or inflamed)	❑	❑	❑	_____ years
k. Rhinitis (eyes, nose and throat reaction to irritants like pollen, fumes, or dust)	❑	❑	❑	_____ years
l. Chronic Bronchitis	❑	❑	❑	_____ years
m. Eczema	❑	❑	❑	_____ years
n. Stomach or intestinal ulcers	❑	❑	❑	_____ years
o. A bowel disorder such as Crohn's disease or colitis	❑	❑	❑	_____ years
p. High blood pressure (sometimes called hypertension)	❑	❑	❑	_____ years
q. Cancer	❑	❑	❑	_____ years
r. Heart disease	❑	❑	❑	_____ years
s. Gastroenteritis (Diarrhea associated with nausea and vomiting)	❑	❑	❑	_____ years
t. Other condition(s) not specified above:	❑	❑	❑	

If **Yes**, please specify:

___________________________ _____ years

___________________________ _____ years

___________________________ _____ years

CUMBERLAND

Section D

This section asks questions about your smoking habits, education and income information, as well possible causes of worries and stress.

1. In your lifetime, have you ever smoked a total of 100 or more cigarettes (about four packs)?

❑ YES ❑ NO

If **YES**, how many years did you smoke? _____ years

On average, how many cigarettes a day did you smoke? _____ cigs/day

2. Do you presently smoke cigarettes:

❑ Daily → How many cigarettes do you smoke each day now? _____ cigs/day

❑ Occasionally → On the days you do smoke, about how many cigarettes do you usually have? _____ cigs/day

❑ Not at all

3. Have you ever lived with another person who smoked on a regular basis?

❑ YES ❑ NO

4. Does anyone in this household smoke regularly inside the house?

❑ YES ❑ NO

5. Have you ever worked with another person who smoked on a regular basis?

❑ YES ❑ NO

6. Have any of these things been a cause of worry or stress for you in the last year? Please check all that apply.

❑ Housing problems
❑ Problems with neighbors
❑ Money problems
❑ Other people's health problems
❑ Air pollution
❑ Unemployment
❑ Work problems
❑ Not feeling safe in your neighborhood
❑ Health problems
❑ Dietary habits
❑ Smoking habits
❑ Exercise habits
❑ Outdoor smells
❑ Drinking habits
❑ Water pollution
❑ Noise
❑ Indoor smells or scents
❑ Traffic fumes

7. What is the language that you first learned at home in childhood? If you no longer understand the first language you learned, please mark the second.

❑ English ❑ French ❑ Other (please specify): ________________

8. Please estimate into which of the following groups your TOTAL household income falls.
   a. Greater than 80,000
   b. 50,000 to < 80,000
   c. 30,000 to < 50,000
   d. 15,000 to < 30,000
   e. Less than 15,000
   f. Prefer not to answer

9. What is the highest degree, certificate, or diploma that you have obtained?
   a. No postsecondary degree, certificate or diploma
   b. Trades certificate or diploma from a vocational school or apprenticeship training
   c. Non-university certificate or diploma (community college, school of nursing, etc.)
   d. University certificate below bachelor's level
   e. Bachelor's degree
   f. University certificate or diploma above a bachelor's degree

10. At present are you (mark all that apply):
   a. Employed full-time outside the home
   b. Employed part-time outside the home
   c. Attending school full or part-time
   d. Unemployed and not looking for work (e.g. disabled)
   e. Unemployed and looking for work
   f. Homemaker
   g. Retired
   h. Other (please specify): ________________

11. Which of the following best describes your occupation?

- ❑ Management
- ❑ Professional (including accountants)
- ❑ Technologist, technician or technical occupation
- ❑ Administrative, financial or clerical
- ❑ Sales or service
- ❑ Trades, transport or equipment operator
- ❑ Occupation in farming
- ❑ Occupation in processing, manufacturing or utilities
- ❑ Other (please specify) ________________

Section E

This section asks you to provide some information about the health of any children who live in your home on a regular basis.

IT IS ONLY NECESSARY THAT THIS SECTION BE COMPLETED IN ONE QUESTIONNAIRE FROM PARTICIPANTS IN THE SAME HOUSEHOLD.

1. When analyzing health, it is important to know whether the participant is pregnant.
   Are you pregnant? ❑ YES ❑ NO ❑ Not applicable

2. How many children (<18 years of age) live in the house? _____ children

   If there are no children in this house, please go to Question 15.

3. What is the gender of each child?

Child 1	Child 2	Child 3	Child 4	Child 5	Child 6
❑ Male	❑ Male	❑ Male	❑ Male	❑ Male	❑ Male
❑ Female	❑ Female	❑ Female	❑ Female	❑ Female	❑ Female

4. What is the age of each child?

Child 1	Child 2	Child 3	Child 4	Child 5	Child 6
___ years	___ years	___ years	___ years	___ years	___ years

5. What is the general health of each child?

Child 1	Child 2	Child 3	Child 4	Child 5	Child 6
❑ Excellent	❑ Excellent	❑ Excellent	❑ Excellent	❑ Excellent	❑ Excellent
❑ Very good	❑ Very good	❑ Very good	❑ Very good	❑ Very good	❑ Very good
❑ Good	❑ Good	❑ Good	❑ Good	❑ Good	❑ Good
❑ Fair	❑ Fair	❑ Fair	❑ Fair	❑ Fair	❑ Fair
❑ Poor	❑ Poor	❑ Poor	❑ Poor	❑ Poor	❑ Poor

6. During the school year, how many nights per week does this child usually sleep in your home?

Child 1	Child 2	Child 3	Child 4	Child 5	Child 6
___ nights	___ nights	___ nights	___ nights	___ nights	___ nights

7. In the last 12 months, has this child had whistling or wheezing in their chest at any time?

Child 1	Child 2	Child 3	Child 4	Child 5	Child 6
❑ YES	❑ YES	❑ YES	❑ YES	❑ YES	❑ YES
❑ NO	❑ NO	❑ NO	❑ NO	❑ NO	❑ NO

8. Has this child ever had a problem with sneezing, or a runny or blocked nose when he or she did not have a cold or flu in the last 12 months?

Child 1	Child 2	Child 3	Child 4	Child 5	Child 6
❑ YES	❑ YES	❑ YES	❑ YES	❑ YES	❑ YES
❑ NO	❑ NO	❑ NO	❑ NO	❑ NO	❑ NO

9. Has this child ever had asthma?

Child 1	Child 2	Child 3	Child 4	Child 5	Child 6
❑ YES	❑ YES	❑ YES	❑ YES	❑ YES	❑ YES
❑ NO	❑ NO	❑ NO	❑ NO	❑ NO	❑ NO

10. Does he or she still have asthma?

Child 1	Child 2	Child 3	Child 4	Child 5	Child 6
❑ YES	❑ YES	❑ YES	❑ YES	❑ YES	❑ YES
❑ NO	❑ NO	❑ NO	❑ NO	❑ NO	❑ NO

11. Has this child had an asthma attack in the last 12 months?

Child 1	Child 2	Child 3	Child 4	Child 5	Child 6
❑ YES	❑ YES	❑ YES	❑ YES	❑ YES	❑ YES
❑ NO	❑ NO	❑ NO	❑ NO	❑ NO	❑ NO

12. Does he/she take any prescribed medication for asthma?

Child 1	Child 2	Child 3	Child 4	Child 5	Child 6
❑ YES	❑ YES	❑ YES	❑ YES	❑ YES	❑ YES
❑ NO	❑ NO	❑ NO	❑ NO	❑ NO	❑ NO

13. Has a doctor ever said this child had hay fever?

Child 1	Child 2	Child 3	Child 4	Child 5	Child 6
❑ YES	❑ YES	❑ YES	❑ YES	❑ YES	❑ YES
❑ NO	❑ NO	❑ NO	❑ NO	❑ NO	❑ NO

14. Has a doctor ever said this child had allergies?

Child 1	Child 2	Child 3	Child 4	Child 5	Child 6
❑ YES	❑ YES	❑ YES	❑ YES	❑ YES	❑ YES
❑ NO	❑ NO	❑ NO	❑ NO	❑ NO	❑ NO

15. Do you have any comments about this questionnaire?

THANK YOU!
